# Uncover rock-climbing fish's secret of balancing tight adhesion and fast sliding for bioinspired robots

**DOI:** 10.1093/nsr/nwad183

**Published:** 2023-06-29

**Authors:** Wenjun Tan, Chuang Zhang, Ruiqian Wang, Yuanyuan Fu, Qin Chen, Yongliang Yang, Wenxue Wang, Mingjun Zhang, Ning Xi, Lianqing Liu

**Affiliations:** State Key Laboratory of Robotics, Shenyang Institute of Automation, Chinese Academy of Sciences, Shenyang 110016, China; Institutes for Robotics and Intelligent Manufacturing, Chinese Academy of Sciences, Shenyang 110169, China; University of Chinese Academy of Sciences, Beijing 100049, China; State Key Laboratory of Robotics, Shenyang Institute of Automation, Chinese Academy of Sciences, Shenyang 110016, China; Institutes for Robotics and Intelligent Manufacturing, Chinese Academy of Sciences, Shenyang 110169, China; State Key Laboratory of Robotics, Shenyang Institute of Automation, Chinese Academy of Sciences, Shenyang 110016, China; Institutes for Robotics and Intelligent Manufacturing, Chinese Academy of Sciences, Shenyang 110169, China; University of Chinese Academy of Sciences, Beijing 100049, China; Department of Histology and Embryology, Basic Medical College, China Medical University, Shenyang 110122, China; Chengdu Institute of Biology, Chinese Academy of Sciences, Chengdu 610042, China; State Key Laboratory of Robotics, Shenyang Institute of Automation, Chinese Academy of Sciences, Shenyang 110016, China; Institutes for Robotics and Intelligent Manufacturing, Chinese Academy of Sciences, Shenyang 110169, China; State Key Laboratory of Robotics, Shenyang Institute of Automation, Chinese Academy of Sciences, Shenyang 110016, China; Institutes for Robotics and Intelligent Manufacturing, Chinese Academy of Sciences, Shenyang 110169, China; Department of Biomedical Engineering, School of Medicine, Tsinghua University, Beijing 100084, China; Emerging Technologies Institute, Department of Industrial and Manufacturing Systems Engineering, University of Hong Kong, Hong Kong 999077, China; State Key Laboratory of Robotics, Shenyang Institute of Automation, Chinese Academy of Sciences, Shenyang 110016, China; Institutes for Robotics and Intelligent Manufacturing, Chinese Academy of Sciences, Shenyang 110169, China

**Keywords:** bioinspired, underwater adhesion, crawling robots, rock-climbing fish, Stefan adhesion, underwater robots

## Abstract

The underlying principle of the unique dynamic adaptive adhesion capability of a rock-climbing fish (*Beaufortia kweichowensis*) that can resist a pull-off force of 1000 times its weight while achieving simultaneous fast sliding (7.83 body lengths per second (BL/S)) remains a mystery in the literature. This adhesion-sliding ability has long been sought for underwater robots. However, strong surface adhesion and fast sliding appear to contradict each other due to the need for high surface contact stress. The skillfully balanced mechanism of the tight surface adhesion and fast sliding of the rock-climbing fish is disclosed in this work. The Stefan force (0.1 mN/mm^2^) generated by micro-setae on pectoral fins and ventral fins leads to a 70 N/m^2^ adhesion force by conforming the overall body of the fish to a surface to form a sealing chamber. The pull-off force is neutralized simultaneously due to the negative pressure caused by the volumetric change of the chamber. The rock-climbing fish's micro-setae hydrodynamic interaction and sealing suction cup work cohesively to contribute to low friction and high pull-off-force resistance and can therefore slide rapidly while clinging to the surface. Inspired by this unique mechanism, an underwater robot is developed with incorporated structures that mimic the functionality of the rock-climbing fish via a micro-setae array attached to a soft self-adaptive chamber, a setup which demonstrates superiority over conventional structures in terms of balancing tight underwater adhesion and fast sliding.

## Teaser

The rock-climbing fish employs micro-hydrodynamics and suction to achieve low friction and high pull-off resistance adhesion, allowing fast crawling while maintaining adhesion. This mechanism inspires an innovative underwater wall-climbing robot design.

## INTRODUCTION

Rock-climbing fish (*Beaufortia kweichowensis*) live on the rocky surfaces of fast-flowing water with a strong flow impact [1]. These fish can quickly slide on large-curvature surfaces and attach to vertical or ceiling surfaces underwater. They can resist a detachment force of 1000 times their own body weight [2–4]. Furthermore, these fish can slide continuously on an underwater surface without attachment and detachment. This mode of strong adhesion and continuous surface movement can provide new inspiration for underwater-robot movements. Similar to gecko research leading to the invention of climbing robots [5,6], as well as novel grasping and manipulation systems [7] that have been proposed for outer-space applications [8], rock-climbing fish may open a new avenue for continuous movement on underwater surfaces, which may be useful for detecting, moving and manipulating in deep-sea environments. However, the underlying principle of the dynamic adaptive adhesion capability of a rock-climbing fish, which has a large adhesion force while achieving simultaneous fast sliding, is still unclear.

Studies on the adhesion and crawling mechanisms of terrestrial creatures have made great progress in recent years [9]. Geckos (e.g. *Gekko gecko*) can hang from ceilings [10,11], beetles (e.g. *Coccinella septempunctata*) can crawl on inclined surfaces [12,13] and tree frogs (e.g. *Litoria caerulea*) can crawl and jump on tree trunks [14,15]. These creatures usually employ molecular attraction [16,17], capillary force [18] *and* mechanical interlocking [19] for adhesion and use highly organized gaits that involve periodic attachment and detachment for climbing [20,21]. They can also jump with fully prepared take-off and landing postures [22]. The successful bionics of terrestrial climbing animals can even be applied to grasp and crawl in outer space. However, due to the changes in physical and chemical properties, these dry adhesion systems may not be applicable in water environments [23,24].

Due to the varied impacts of underwater dynamics, reattachment after detachment becomes extremely challenging. This has been a great challenge with regard to the development of underwater robots and vehicles. However, most aquatic species employ sucking disks that can attach themselves to substrate surfaces with little movement [23]. The remora (e.g. *Echeneis naucrates*) uses a suction cup to attach to other moving objects but is unable to move by itself when adhering to objects due to having a large preload [8,25,26]. The northern clingfish (e.g. *Gobiesox maeandricus*) uses a negative pressure chamber formed by the pectoral fin and pelvic fin to adhere to rocks and other surfaces and can only wriggle to move forward [27]. Octopuses (e.g. *Octopus vulgaris*) use multiple feet and suction cups that are redundant and employ sequential adhesion detachment to achieve slow crawling [28,29]. Waterfall-climbing gobies (e.g. *Sicyopterus spp*) can climb waterfalls using an ‘inching’ behavior, in which an oral sucker is cyclically protruded and attached to the surface [30–32]. These creatures have strong adhesion abilities but poor movement abilities. The adhesion-sliding movement is challenging because strong adhesion and fast sliding appear to contradict each other, and detachment is required in most cases before moving.

Based on the climbing gait of the rock-climbing fish, a biomimetic crawling robot (out of water) was designed. The robot can crawl on walls through a combination of two suction cups and four friction pads [33]. Through bionic remora adhesion, an aerial-aquatic robot was designed. The robot can not only adhere to surfaces in both aerial and aquatic environments via a suction cup, but also achieve cross-media movement with a quadrotor aircraft [26]. By combining pectoral fin swimming and a suction cup, a biomimetic robot was designed to achieve hitchhiking underwater [34], and another remora-inspired robot can achieve sliding on the bottom surface underwater and strong adhesion when stationary [35]. However, few robots have shown that they can adhere and crawl on bottom, side and ceiling surfaces.

Rock-climbing fish (*B. kweichowensis*) use their whole bodies as suction cups for adhesion [2] and do not need to detach from surfaces when sliding continuously at a speed of 7.83 body lengths per second (BL/s) (Fig. S1). This excellent ability to balance large adhesion and fast sliding has always been puzzling in the scientific community. Zou *et al.* proposed that microbubbles on the ventral side help seal the suction disc [36]. Chuang *et al.* suggested three possible mechanisms: (i) setae enhancing the friction force of substrates, (ii) setae interlocking with irregularities and (iii) setae sealing the edge to prevent leakage [4]. The excellent motion ability achieved by balancing large adhesion and fast sliding is of great scientific interest and is potentially significant in emerging engineering applications. However, the underlying mechanism for simultaneous adhesion and sliding is still a mystery.

In this paper, it was found that negative pressure was the force contributing the main resistance to pull-off, and the micro-setae on the pectoral and ventral fins of the fish played a key role in forming a negative chamber. At micro-setae, there is an adhesion force of 0.1 mN/mm^2^, and this force is produced by the microfluidic hydrodynamic interaction between the setae array and substrate, known as the Stefan force. This tiny adhesion force not only ensures that these fish have a low preload force, thus generating a small amount of friction when sliding on surfaces, but also assists in the overall body conforming to the surface to form a negative pressure chamber when detachment force is applied. Inspired by the unique adhesion-sliding mechanism and corresponding microstructures contributing to the underlying mechanism, a soft bionic robot with a corresponding micro-setae structure was developed in this study. The robot, which is named ‘Climbot’, can tightly adhere to our sailing ship model in a river and can be made to crawl continuously at the bottom of the ship. This skillfully balanced adhesion-sliding mechanism may offer new ideas with regard to realizing underwater on-surface movement and bring new inspiration to the design of underwater adhering-moving robots.

## RESULTS AND DISCUSSION

### The sliding ability, mechanism and bionics of rock-climbing fish

**Figure 1.**
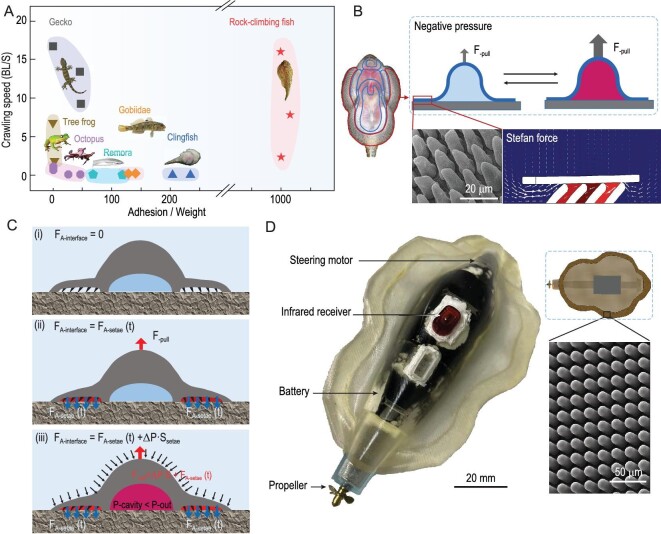
Adhesion and crawling ability, and mechanism of the rock-climbing fish and biomimetic prototype design. (A) Among the adhering species, the rock-climbing fish can generate great adhesion force with amazing crawling ability underwater. (B) The setae array on the edge region of the suction cup conforms to the substrate and forms a sealing chamber through the Stefan force; the inner cavity of the suction cup presents a pressure change due to the volume change when the pull-off force is imposed. (C) The mechanism for generating negative pressure adhesion: when the pull-off force occurs, the micro-setae at the edge of the suction cup are kept in contact with the substrate by force ‘F_A-setae_’ due to hydrodynamic interactions, and the suction cup keeps sealed during the pull-off process. However, the suction cup deforms to increase its volume and decrease its inner pressure, generating negative pressure adhesion to resist the pull-off force throughout the process. (D) A bionic underwater crawling/climbing fish named Climbot was designed based on the adhesion mechanism of the fish, and can crawl at a maximum speed of 3.7 BL/S and has an adhesion force of 25.67 ± 2.81 N.

Rock-climbing fish have been reported to have remarkable adhesion ability and sliding speed [3,27,37–39] (Fig. 1A). Two types of forces contribute to balancing the pull-off force: the Stefan force on the setae, which is distributed on the edge of the suction cup, and the suction force at the abdomen (Fig. 1B) [2]. When no pull-off force is imposed, the fish is in contact with the substrate with only a little interface force. Once the pull-off force is applied, the micro-setae on the edge of the suction cup generate a Stefan force of 0.1 mN/mm^2^ due to hydrodynamics, causing the edge to conform to the substrate, which forms a sealing chamber. The volume of the chamber increases under the pull-off force, which decreases the pressure of the chamber. The pressure difference resists the pull-off force (Fig. 1C). The interface friction is minimal due to the limited Stefan force, so the fish can slide rapidly. Moreover, the fish can adapt spontaneously to the pressure of the suction cup to adjust the friction and adhesion force in order to realize on-surface movements. Inspired by this unique adhesion and sliding mechanism, Climbot was designed. The body of the robot was 3D printed with soft material agilus 30. The micro-setae array was manufactured using replica modeling technology with a lithographic master and mounted around the margin of the body. Three motors were used for driving, steering and pressure adjustment. The robot was remotely controlled by infrared (Fig. 1D).

### The synergistic process of adhering and sliding under propulsion

**Figure 2.**
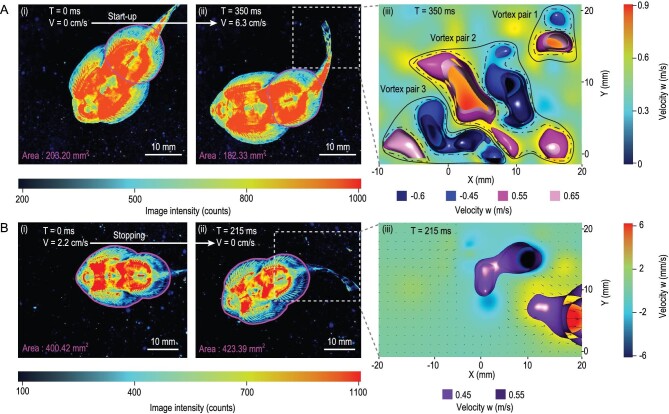
The adhering-sliding synergistic process is shown by the contact zone and propelling flow field image during starting and stopping. (A) The starting movement of the rock-climbing fish. (i) T = 0 ms, initial state, the fish stay static; (ii) T = 350 ms, start-up is realized via cooperation between adhering and swinging the caudal fin. (iii) The flow field after the caudal fin swings at T = 350 ms, containing three swings. (B) The stopping movement of a rock-climbing fish. (i) T = 0 ms, initial state, the fish is about to brake during sliding. (ii) T = 215 ms, stopping is realized by increasing adhesion via enlarging the suction cup area and the forepart of the suction cup first adhering to the substrate, followed by the posterior half. (iii) The flow field around the caudal fin at T = 215 ms.

To investigate how to realize fast switching and crawling based on this adhesion mechanism, the switching process of adhesion and crawling was visualized through frustrated total internal reflection (FTIR) combined with 4D particle tracking velocimetry (PTV). The flow field as the fish starts to slide and stops moving, and the attachment area between the disc and substrate, were obtained, as shown in Fig. 2.

During the start-up stage (Fig. 2A-i and A-ii), the rear suction cup of the rock-climbing fish has an area of 182.33 mm^2^ at T = 350 ms, as Fig. 2A-ii shows, which is 10.27% less than that in Fig. 2A-i (203.20 mm^2^ at T = 0 ms). A smaller contact area has a smaller friction force with the surface. During this process, the front suction cup of the rock-climbing fish adheres and anchors while the rear suction cup releases and reduces friction. Accompanied by the swing of the tail fin, the entire body forms an arch along the axis, achieving start-up. When t = 350 ms, the tail fin flapped three times and generated three pairs of vortices (Fig. 2A-iii), and during the whole start-up process, the fish was always attached to the surface (Fig. 2A-i and A-ii).

At the stopping stage, the rock-climbing fish gradually increased its adhesion force and stopped on the surface, and the tail fin made passive deformations under the action of the flow field to attenuate the force of the flow field (Fig. 2A-iii). From the FTIR data results in Fig. 2B-i and B-ii, it can be seen that the contact area (high image intensity value area) of the suction cup became sharper compared to Fig. 2A-i and A-ii, and the maximum light intensity value of the suction cup from the FTIR contact image is 980 counts in Fig. 2A-i and A-ii, while Fig. 2B-i and B-ii show the maximum light intensity value increases to 1100 counts. This indicates that the abdomen of the fish is concave and that the suction cup is in an active suction-adhering state. Additionally, the area of the suction cup of the fish in Fig. 2B-ii is 5.4% larger than that in Fig. 2B-i. By expanding the body, thus increasing the contact area, the fish has increased friction with the substrate.

By examining the adhesion and crawling processes (see Fig. 2 and Movie S1) we found that: (i) when the rock-climbing fish crawls or adheres to the wall, it always maintains an attachment to avoid being separated from the surface by the impact of water flow during the switching of adhesion state; (ii) when it stops, the fish increases the adhesion force by contracting its abdomen to realize anchoring, which means the adhesion force can be regulated by adjusting the negative pressure; and (iii) during start-up, the fish switches from the adhering anchoring state to the crawling state without detaching from the surface.

### Tunable negative pressure adhesion

To investigate the adhesion force found in different situations, we defined four states of the fish: the static state, the crawling state, the defending state and the pull-off state (Fig. S2A and B). In the static state, the suction pressure varies periodically. The peak value of the pressure difference is 113.11 ± 19.02 Pa (*n* = 15) (Fig. S2C). During crawling, the sequential changes in the suction pressure were acquired by four pressure sensor sequences attached to the substrate, and negative pressure of the chamber appeared along the sliding route (Fig. S2D), which indicates that the sealing chamber was formed dynamically and adaptively during the sliding process, and the pressure difference was 276.27 ± 59.82 Pa (*n* = 15). As such, it is maintained in a sealing state by the setae to generate negative pressure. Thus, the fish can simultaneously resist large pull-off forces during crawling. When the fish are under hydrodynamic impact, they switch to a defensive state, conforming the setae on the pectoral and ventral fins to the substrate to form a sealing chamber and shrinking the abdomen to enlarge the volume of the sealing chamber. The pressure difference in this state reaches 596.79 ± 150.8 Pa (*n* = 15), increasing the adhesion force (Fig. S2E).

When an external pull-off force was applied, the maximum detachment force and suction pressure of the fish were measured at the same time. The sucking pressure was close to zero (dead fish) when there was no pull-off force. When the fish was pulled, the difference between the maximum pressure of the suction cup and the outer pressure was 58.07 ± 12.85 kPa (*n* = 6). The dead fish could also resist a large pull-off force, which implies that the adhesion force can be generated by the structures (see Movie S2 from Fig. S2F).

### Morphology of the suction cup and adhesion ability

**Figure 3.**
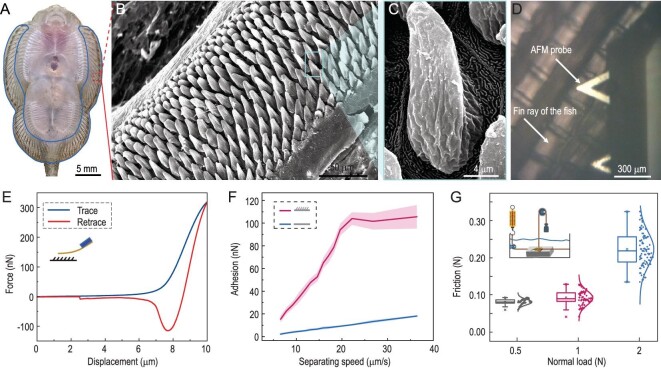
Characterization of the morphology of the rock-climbing-fish suction cup, and its adhesion characteristic. (A) Bottom view of the fish suction cup. (B and C) Setae morphology. (D) Microscope view of setae adhesion measurement with atomic force microscope (AFM). (E) Force-displacement curve of setae adhesion measurement with AFM shows that there is no jump in the trace curve, indicating that there is no attraction force such as van der Waals force or electrostatic force, only adhesion force. (F) Variation of adhesion with approach velocities of AFM tip-less cantilever; the adhesion force increases with the AFM probe speed. The retrace curve shows the adhesion force from setae. The trace curve shows the adhesion force from a flat surface (petri dish). (G) The friction of fish under different pulling forces. The pulling forces are provided by the normal load of weights.

To determine the formation conditions of the sealing cavity, we studied the morphology of the suction cup and the adhesion force of these structures. Through microstructure characterization, three types of microstructures dividing the abdomen into three areas were found (see Fig. S3). The pectoral and ventral fins serve as the outer edges of the suction cup (Fig. 3A). As shown in Fig. 3B and Fig. S3B, there are tongue-like and cone-like setae distributed on the fin ray, with a density of 9000–10 000 per square millimeter, a diameter of 2–6 microns and a height of 12–14 μm. This structure, made of ‘unculi’, is composed of keratin or dead cells. It only exists in the order Ostariophysi and often works with adhesion devices [40]. Dense transparent vesicles are distributed at the junction of the abdomen, pectoral and pelvic fins, and the outer edge of the mandible. The vesicles are (Fig. S3D) on the inner side of the abdomen, and there is a hexagonal-textured structure (see Fig. S3E).

According to the contact splitting effect, the actual contact of the suction cup of the fish with the substrate can be regarded as the accumulation of numerous small flat contacts. We used a tip-less probe with AFM to measure the force-displacement curves of the three microstructures in the suction cup of the rock-climbing fish, and it was found that there was notable adhesion force only in the area of the micro-setae (see Fig. S4). The micro-setae have greater stiffness, and the Derjaguin-Muller-Toporov (DMT) modulus is three orders of magnitude higher than that of surrounding tissue (see Fig. S5). Therefore, the soft tissue can enhance the lip ring's deformation and adaptability, better fitting the substrate. For the harder setae, the deformation of the setae is smaller, and the recovery of shape is faster. This makes it less likely that a large deformation of micro-setae will occur when the suction cup is attached to the substrate. The adhesion force of micro-setae is not caused by the accumulation of single seta adhesion. Figure S6 shows that there is no significant adhesion force when four or five micro-setae are in contact with the tip-less probe. However, when more micro-setae were in contact with the probe, there was a notable adhesion force generated, which indicates that a set of setae working cohesively forms the adhesion force. The force-displacement curve is shown in Fig. 3E. No jump-in in the trace curve indicates no attraction force, such as van der Waals or electrostatic forces. A 112.83 ± 6.23 nN (*n* = 51) adhesion force was observed in the retraction curve. The setae were the key structure for the formation of the sealing chamber, which can generate a hydrodynamic adhesion force to ensure that the whole body conforms to the surface when detachment force is applied.

By applying different separating velocities to the AFM probe, the adhesion force of the micro-setae increased significantly with increasing separation velocity (Fig. 3F). Taking the petri dish as a substrate for the control group, the change in adhesion force on a flat surface with increasing separation velocity was measured. It was found that the increase in adhesion force with an increase in separating velocity was not obvious, indicating that the generation and characteristics of this kind of adhesion force had a great relationship with the spatial topology of micro-setae, and the force is also directly related to the dynamic interaction process of micro-setae, adhesion surfaces and water.

The friction force of the fish under different pull-off forces on a smooth surface underwater was obtained. The friction force is 81 ± 8 mN under a 0.5 N pull-off force (92 ± 19 mN under 1 N, 223 ± 45 mN under 2 N), which is much smaller than that of geckos and remora (the friction of a gecko under zero preload reaches 3.5 N [41], and for remora the backward frictions on a smooth surface are 20.93 N to 35.62 N [8], as shown in Fig. 3G). Because the contact stress and friction force at the interface can be very small during adhesion, the fish can slide fast when adhering.

### Theoretical analysis of the setae adhesion mechanism

**Figure 4.**
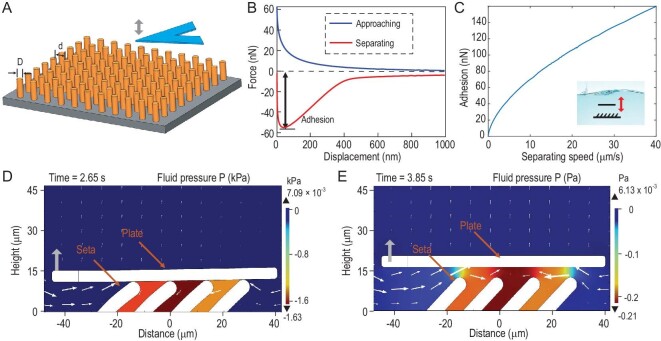
Simulation of setae hydrodynamic adhesion. (A) Schematic for the topographical features and the adhesion testing process. (B) The analytic solution of the force-displacement curve is obtained from Equation S5 in the online supplementary data. (C) The analytic solution of adhesion vs. separating speed obtained from Equation S5. (D) The simulation result of adhesion between setae and a moving plate. When the plate is just separating from the four setae, and a negative pressure area is generated (pressure reaches −1.63 kPa), this negative pressure causes the adhesion force. (E) The plate lifts to 5 μm, and the pressure goes to −0.2 Pa.

To further analyze the formation mechanism of the adhesion force of micro-setae under hydrodynamic interaction, we separated two plates in an incompressible Newtonian fluid. According to the adhesion measurement experiment shown in Fig. S6, a numerical simulation was set up as shown in Fig. 4A. According to the set-up in the measurement of setae adhesion force with AFM (Fig. 3D–F), we set the maximal separation as 1000 nm, the minimal separation as 1 nm (approaching limit), the ramp speed of the AFM probe as 4 μm/s, and the coefficient of the elasticity of the AFM probe as 0.24 N/m. We also used a mixed fourth- and fifth-order Runge–Kutta method to solve Equation S5 in the online supplementary data numerically and obtained theoretical predictions for the hydrodynamic forces.

The hydrodynamic repulsion when the AFM probe is driven toward the micro-cylindrical array is predicted by the approaching curve in Fig. 4B; the adhesion when the AFM probe is driven and separated from the micro-cylindrical array is described by the separating curve. This numerical solution has the similar force-change trend as Fig. 3E and the same order of magnitude in adhesion.

We also investigated adhesion as a function of separating speed. Based on Equation S5, we obtained the theoretical prediction of adhesion with different separation speeds, as shown in Fig. 4C. In the experiment, we found that the adhesion force increased non-linearly with increasing separation speed, while on the flat substrate, the adhesion force increased linearly and slowly with separation speed, as shown in Fig. 3F. In the numerical simulation, the results are similar to the experimental results (Fig. 4C); that is, the adhesion force of the micro-column increases non-linearly with the separation velocity.

To better understand the mechanism of the micro-setae adhesion force, the hydrodynamic behavior of the micro-setae in water was simulated by using a fluid–solid coupling module in COMSOL 5.5. In the geometric model, four micro-columns with a diameter of 5 μm, length of 12 μm, inclination angle of 45° and spacing of 5 μm were set up, and the rectangle with 90 μm length and 4 μm width moved downward at a speed of 10 μm/s. The Young's modulus of the microcolumns was E = 200 MPa. The Poisson's ratio of the microcolumns was nu = 0.4, the Young's modulus of the rectangle plate (silicon) was E = 170 GPa and the Poisson's ratio of the rectangle plate (silicon) was nu = 0.28. The full coupling transient solution of the flow field and multi-body dynamics was carried out to visually characterize the negative pressure region and dynamic change process between the micro-column and the flat plate due to fluid dynamics. The arrow on the drawing indicates the direction of the movement of fluid. In the area between the micro-column and the flat plate, due to the viscous characteristics of the fluid, a low-pressure region (Fig. 4D) is formed between the micro-columns. The plate separates from the four setae and generates a negative pressure area (pressure reaches −1.63 KPa). This negative pressure causes an adhesion force, and when the plate lifts to 5 μm, the pressure goes to −0.2 Pa (Fig. 4E).

### Setae array fabrication and adhesion force

**Figure 5.**
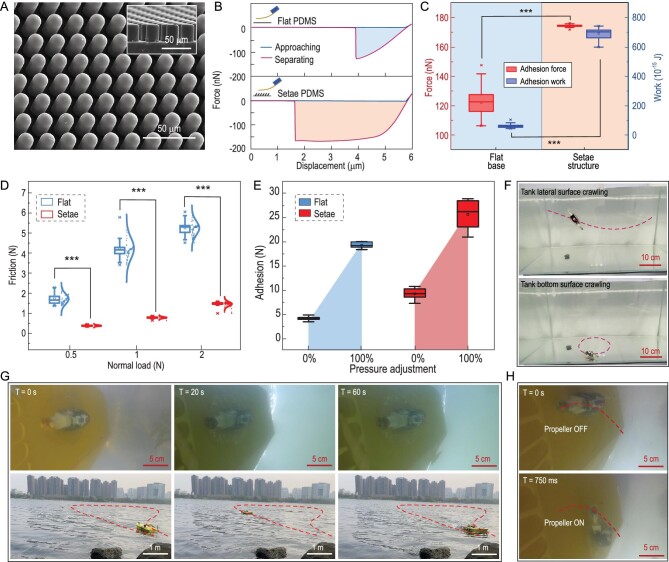
Adhesion ability of Climbot, and outdoor experiments. (A) Biomimetic setae. The length of the biomimetic setae is 15 μm, and the cylinder setae diameter is 12 μm. (B) The force-displacement curve of setae and the flat substrate; the biomimetic setae show a better adhesion performance than the flat base. (C) The adhesion force and work contrast the flat base and setae structure; the adhesion force of the setae structure is more stable than the flat base, and the adhesion force of the biomimetic setae is 42.7%. The adhesion work of the biomimetic setae is 11.9 times stronger than that of the flat base. (D) The friction comparison between a bionic underwater crawling robot with a setae structure, and one without a setae structure, with a different load, shows that the former has lower friction. (E) An adhesion comparison between a bionic underwater crawling robot with a setae structure and one without a setae structure shows that the former has higher adhesion. (F) Climbot can slide on the bottom lateral surfaces of the tank. (G) Climbot attached tightly to a model ship while the vessel was sailing on a river. (H) Climbot was directed to move on the bottom surface of a model ship (Mann−Whitney-Wilcoxon test, *** *P* < 0.001).

To mimic the setae array of the fish and its hydrodynamic interaction, we fabricated a Polydimethylsiloxane (PDMS) micro-setae array through replica modeling technology. The diameter of the setae is 10 microns, with a height of 30 μm, as shown in Fig. 5A. Force-displacement curves are shown in Fig. 5B. The adhesion of the micro-setae is 174.5 ± 0.68 nN (*n* = 90), which is 42.7% higher than that of the flat base (122.2 ± 7.86 nN (*n* = 90)). The flat base and the bionic micro-setae are made of the same material.

During the adhesion measurement on the flat base, the cantilever beam makes contact with the substrate and then retracts. The adhesion force of the cantilever beam on the flat substrate increases rapidly with a large slope. When the maximum adhesion force is reached, the cantilever beam and substrate immediately separate, and the adhesion distance between the cantilever beam and flat substrate is ∼1 μm (see the top half of Fig. 5B). During the adhesion measurement on the setae array structure, we found that in the process of cantilever withdrawal, the adhesion force first increased with a large slope, then increased slowly, and then remained in a relatively flat range. After the cantilever lifted 4.9 μm, it separated from the base. The adhesion distance between the cantilever beam and setae array structure is ∼4.6 μm, which is 4.6 times that of the flat base. The long adhesion distance of the setae array structure plays a key role in maintaining adhesion stability, adaptability to the surface and dissipation of the detachment energy (see the lower half of Fig. 5B).

The adhesion force and work of the multipoint force-displacement curves were analyzed (Fig. 5C). As shown in Fig. 5C, the adhesion of the setae array is very centralized, and the adhesion force changes in a small range. However, in a flat base, the error is large, and the variation range is large (the box represents 25%–75% of the samples), indicating that the adhesion of the flat base is unstable. This is because the flat substrate has poor adaptability to the surface of the cantilever beam, while the setae array structure can adapt better to the surface, ensuring more stable contact with the substrate and therefore allowing the adhesion force to be relatively stable. Additionally, the adhesion work of biological setae was 11.9 times greater than that of the flat base (Mann−Whitney-Wilcoxon tests were adopted). All tests were two-sided, and a *P* value less than 0.05 was considered statistically significant. The excellent adaptability of the setae is the key to ensuring reliable and stable adhesion.

### A bioinspired crawling robot based on the adhering crawling mechanism

Inspired by the above adhesion mechanism, we developed an underwater soft crawling robot (see Movie S3); the body of the robot was fabricated from a soft material (agilus 30) using a multi-material 3D printer. The micro-setae structure was fabricated via lithography-assisted replica molding micromachining technology, with PDMS. The piston was situated in the abdomen of the Climbot and driven by a motor to adjust the pressure of the suction cup. A propeller was installed at the tail to generate the thrust. Gears and a motor were installed at the head for left and right thrust steering.

When adjusting the pressure of the sucking disk from minimum to maximum, the adhesion force of the Climbot ranged from 9.27 ± 1.24 N to 25.67 ± 2.81 N (*n* = 6), and the adhesion force of the non-setae Climbot ranged from 4.20 ± 0.42 N to 19.30 ± 0.59 N (*n* = 6). This showed that the setae structure could significantly improve the adhesion force of the robot (Fig. 5E).

Moreover, as shown in Fig. 5D, the friction of the Climbot with setae is much smaller than that of the Climbot without setae under different preloads. The Climbot with micro-setae has a higher adhesion force and lower friction force, which indicates that micro-setae are key to realizing tight adhesion and fast crawling.

By controlling the suction force and propeller, Climbot can crawl straight ahead or in a circle on the fish tank's surfaces (i.e. the bottom surface, lateral surface and ceiling surface) (see Movie S4), and Movie S4 shows that the maximum speed reached 3.7 BL/s on the ceiling surface. To test the adhering holding capacity and impact resistance of Climbot, Climbot adhered to the bottom surface of the ship to realize hitchhiking. After controlling the ship's sailing direction in the river for one minute at a speed of 1.5−2 m/s, the wave height was 0.1 m to 0.2 m. Climbot was still firmly attached to the bottom of the ship (Fig. 5G). Finally, by turning on the propeller, we controlled Climbot to crawl on the bottom surface (see Movie S5), and fast switching between adhering and crawling under this adhesion mechanism was achieved (Fig. 5H).

## CONCLUSION

In this study, the adhesion mechanism of rock-climbing fish was explored. This adhesion action is a process of the ingenious utilization of water by the suction-cup structure of rock-climbing fish. First, due to hydrodynamic interactions with the substrate, the micro-setae can dynamically and adaptively form a sealed cavity with a small friction resistance while sliding. Second, water is nearly incompressible; thus, the negative pressure chamber will not have a large deformation in the process of resisting the pull-off force, which can maintain the stable shape of the suction cup. The dependence on water indicates that the hydrodynamic adhesion on setae plays a key role in the formation of negative pressure. The tiny adhesion force ensures that the fish has a low preload force, thus generating low friction while sliding. Additionally, it also ensures that the whole body conforms to the surface to form a negative pressure chamber when a pull-off force is applied. The combination of the setae's Stefan force and whole-body suction force allows the fish to remain balanced, achieve fast sliding and maintain tight adhesion simultaneously. Inspired by this unique mechanism, an underwater robot is developed with incorporated structures that mimic the functionality of the rock-climbing fish by fabricating a micro-setae array and attaching it to a soft self-adaptive chamber. This set-up has superiority over conventional structures in terms of balancing underwater tightness adhesion and fast sliding. This ‘skillfully balanced’ adhesion-sliding mechanism may offer new ideas with regard to realizing underwater on-surface movement and bring new inspiration to the design of underwater adhering-moving robots.

## METHODS

Detailed methods and materials are given in the online supplementary data.

## Supplementary Material

nwad183_Supplemental_Files

## Data Availability

All data are available in the main text or the supplementary materials. Additional data related to this paper are available at https://doi.org/10.7910/DVN/NRKAP7.
