# Supplementary material for: Uncover rock-climbing fish's secret of balancing tight adhesion and fast sliding for bioinspired robots
Source: Natl Sci Rev. 2023 Jun 29;10(8):nwad183. doi: 10.1093/nsr/nwad183 (PMC10408705; doi:10.1093/nsr/nwad183)
Supplement: nwad183_Supplemental_Files [file nwad183_supplemental_files.zip › Supplementary data.pdf]

Supplementary Materials for

Uncover rock-climbing fish's secret of balancing tight adhesion and fast sliding for bioinspired robots

Wenjun Tan *et al.*

*Corresponding author. Email: lqliu@sia.cn (L. L.), zhangchuang@sia.cn (C. Z.).

This PDF file includes:

MATERIALS AND METHODS

Figs. S1 to S6

Tables S1 to S2

Other Supplementary Materials for this manuscript include the following:

Movie S1. Contact status with the substrate during sliding and adhering of rock-climbing fish by FTIR.

Movie S2. Adhesion performance of the rock-climbing fish.

Movie S3. Rock-climbing fish and bio-inspired underwater climbing robot "Climbot".

Movie S4. Adhesion and crawling performance of Climbot: on water tank surfaces

Movie S5. Adhesion and crawling performance of Climbot: on the bottom ship surface in a river

MATERIALS AND METHODS

Sample preparation

All experiments and surgical procedures were approved by the Animal Use and Care Committee at Chengdu Institute of Biology, Chinese Academy of Sciences, which complies with the National Institutes of Health Guide for the Care and Use of Laboratory Animals (AUCC number: CIBDWLL2022032). All efforts were made to minimize the number of animals used and their suffering.

We collected 20 rock-climbing fish (*B. kweichowensis*) in the field, ranging from 4–6 cm in body length, and raised them in a 60×100×50 cm fish tank. The fish tank was equipped with a filter device, an air pump, and a thermostat. The temperature was maintained at 22 ±1 ℃, and the fish were fed every day with multiple algae wafers. All experiments were completed within six hours after sacrifice to keep adhesion performance.

Visualization of adhering and sliding synergistic process under propelling

Seeded hollow glass beads at a diameter of 10 μm in the 280×180×220 mm water glass tank allowed the fish to crawl on the tank wall, which was recorded continuously at the same time through four cameras with specific angles. The 4D-PTV system based on the "shake the box" algorithm (MiniShaker, Lavision Corporation, Germany) solved the trajectory of particle movement in the flow field and obtained the information of the vortex in the flow field after postprocessing of the algorithm. This system lighted the glass tank wall in parallel and took pictures with the camera perpendicular to the wall. FTIR data were obtained simultaneously, and the information on the contact state between the fish and substrate could be analyzed.

Microstructure characterization of rock-climbing fish sucking disc

The fish were immersed in 0.5 g/L MS-222 (Sigma Chemical, USA) for 15 minutes. The suction cup was washed with phosphate buffer solution (Sigma Chemical, USA) for 10 minutes, and the junction zone of the abdomen and fin was observed in phase difference mode with an Eclipse Ti microscope (Nikon, Tokyo, Japan). Another dissected suction cup tissue was fixed with 2.5% glutaraldehyde (EM Grade) (Solarbio, Beijing Co. Ltd.) for 24 hours. To reduce the damage of glutaraldehyde to the enzyme activity of tissue cells, fixation was carried out at 4 ℃. All utensils, instruments, and glutaraldehyde fixatives were precooled. We rinsed the fixed tissue with phosphate buffer solution (Sigma Chemical, USA) three times for 10 minutes each time. Gradient dehydration: 10%, 30%, 50%, 70%, 90%, and 100% ethanol were used for gradient dehydration for 15 minutes each time, and 100% ethanol was used three times. Replacement: The samples were treated with a 1:1 alcohol and tert-butyl alcohol (Aladdin, (Shanghai) Co. Ltd) mixed solution for 30 minutes and then treated with pure tert-butyl alcohol twice for 20 minutes each time. The container holding the samples was put into a refrigerator at 4 ℃ for 15 minutes, and the liquid tert-butyl alcohol turned into solid ice. The frozen samples were put into a vacuum freeze dryer (Fd-2, Beijing Boyikang Experimental Instrument

Co., Ltd., China) at -80℃ for 12 hours after solid-state tert-butyl alcohol sublimation, and the dried sample was obtained. The dried sample was placed in a vacuum spraying apparatus, sprayed with gold to a thickness of 25 nm, and then subjected to scanning electron microscopy (SEM; Evo ma10, Zeiss, Inc., Germany) for scanning observation.

Micro setae force curve measurement

The fish were immersed in 0.5 g/L MS-222 (Sigma Chemical, USA) for 15 minutes. Then, the pectoral and pelvic fins were dissected and washed with phosphate buffer solution (Sigma Chemical, USA) three times for 10 minutes each time. The fins were bonded to the substrate of the culture dish with ultraviolet curable glue (NOA63, Edmund Optics), and phosphate-buffered saline (PBS) was added. The force curve was measured by using the tip-less probe (NP-O10-B, spring constant of 0.24 N/m, width of 40 μm, length of 200 μm) in the AFM (Dimension Icon, Bruker Corporation, USA) contact in liquid mode. Under ramp speeds of 1-22 μm/s, the force curve of the micro setae was obtained, and the relationship between adhesion force and separating speed was obtained.

Manufacturing the setae array

Through deep silicon etching technology, a ring pattern silicon substrate mold with a width of 10 mm was obtained. The ring pattern was obtained by fitting the outline of the contact area between the fish and the substrate and then doubling the length and width. The ring pattern silicon mold was filled with micro pits with a diameter of 12 μm, spacing of 12 μm, and depth of 15 μm. Sylgard 184 (Dow Corning, USA) prepolymer and crosslinker at a ratio of 10:1 were mixed, degassed, and poured onto a silicon mold. After being victimized for 0.5 hours, the silicon mold with the mixture was sent to a baked oven for 2 hours at a temperature of 80℃. After natural cooling, the film was removed to obtain a ring pattern with a micro setae array.

Manufacturing Climbot

The main body of Climbot was made using a multi-material 3D printer (J735, Stratasys, USA) with the soft material agilus30 clear, and we set the Shore hardness to 30 HA, which is near the hardness of the fish body**.** Climbot is 110 mm long, 80 mm wide, and 25 mm high. It is modeled according to the rock-climbing fish with a scale ratio of 1:2. The robot was fine-tuned to install batteries, printed circuit boards (PCBs), and motors, among many other engineered parts. The weight of the robot is 65 grams. After removing the supporting material, we bound the PDMS film with the setae structure, which was obtained before the edge of the suction cup of the printed main body. We also installed the motor, piston structure, propeller, infrared receiver, PCB circuit board, and lithium battery at the corresponding position and finally sealed it. After the waterproof test, Climbot was able to function after being charged.

Setae array characterization and adhesion measurement

The PDMS film with a micro setae array was placed in a vacuum spraying apparatus, sprayed with gold to a thickness of 25 nm, and subjected to scanning electron microscopy (SEM; Evo ma10, Zeiss, Inc., Germany) to obtain morphological information. Another PDMS film with a setae array was bonded to the substrate of a culture dish with

ultraviolet curable glue (NOA63, Edmund Optics), and PBS was added (keeping the same setae measurement of the fish). The force curve was measured using the tip-less probe (NP-O10-B, spring constant of 0.06 N/m, width of 40 μm, and length of 200 μm) in the AFM (Dimension Icon, Bruker Corporation, USA) contact in liquid mode.

Setae array hydrodynamic adhesion force model

When two surfaces approach each other in an incompressible Newtonian fluid, a hydrodynamic repulsion force will be produced, and when they are separated from each other, an adhesion force will be produced, which can be described by the Reynolds equation in lubrication approximation. When the Navier Stokes equation (Eq. 1) is simplified in the lubrication approximation, fluid inertia, and body force are ignored. The theory also assumes that there is a no-slip boundary condition at the fluid-solid interface[1].

$$\rho \frac{dV}{dt} = \rho g - \nabla p + \mu \nabla^2 V \tag{1}$$

When the fish approach and separate from the substrate surface underwater, they are greatly influenced by hydrodynamics, especially at the microscale. When the distance between two plates is very close, the hydrodynamic force is dominant, and the normal hydrodynamic interaction between two disks is also known as Stefan adhesion[2] (Eq. However, on a microscale, fluid dynamics are very complex. In this paper, we simplified the model of micro setae and aimed to use this model to explore the mechanism and process of the adhesion force of micro setae under water. Ignoring the deformation and inclination angle of the micro setae, the micro setae are equivalent to a vertical micro cylinder that is rigid and nondeformable, and the tongue or conical micro setae are equivalent to a cylinder with an approximate diameter.

$$F_{stefan} = \frac{3}{2} \pi \eta R^4 \frac{\dot{h}}{h^3} \tag{2}$$

When the surface separation (h) is much smaller than the period of the surface microstructure pillar (L) (h <<L), the hydrodynamic forces (F_H) within Reynolds' lubrication theory are given by(*43,44*):

$$F_H = -\frac{6\pi\eta R_G R_H}{h} \frac{dh}{dt} \tag{3}$$

where η is the fluid viscosity, in our case, 25℃ in water, the value is 0.8949×10^{-3} Pa s, $\frac{dh}{dt}$ is the rate of change of the separation, and $R_H = 2R_1R_2 / (R_1 + R_2)$ and $R_G = (R_1R_2)^{1/2}$ are the harmonic and geometric means of the radii of the two cylinders in the crossed-cylinder geometry.

In our experimental setup, we used AFM with a tipless probe to obtain the force curve as a plate approached and separated from the setae. Thus, the hydrodynamic forces (F_H) measured with AFM can be presented by:

$$F_H = k(h - h_0 - vt) \tag{4}$$

where k is the coefficient of elasticity of the AFM probe, v is the trace or retrace velocity of the AFM probe, and h_0 is the initial separation.

By combining Eq. 3 and Eq. 4, we have a first-order differential equation (Eq. 5) to describe the quasi-static force balance between the hydrodynamic drainage forces and the AFM probe restoring spring force.

$$k\left(h - h_0 - vt\right) = -\frac{6\pi\eta R_G R_H}{h}\frac{dh}{dt} \quad (5)$$

Statistical analysis

Normality tests were performed before the formal analyses. If the data were normally distributed, the Student's t-test was used to examine the significance of differences between the two groups. Otherwise, Mann–Whitney-Wilcoxon tests were adopted. All tests were two-sided, and a P value less than 0.05 was considered statistically significant. All statistical analyses in this study were performed using SPSS 20.0 software (IBM Corp, USA). Data are listed as the means ± SDs (standard deviation).

Fig. S1.

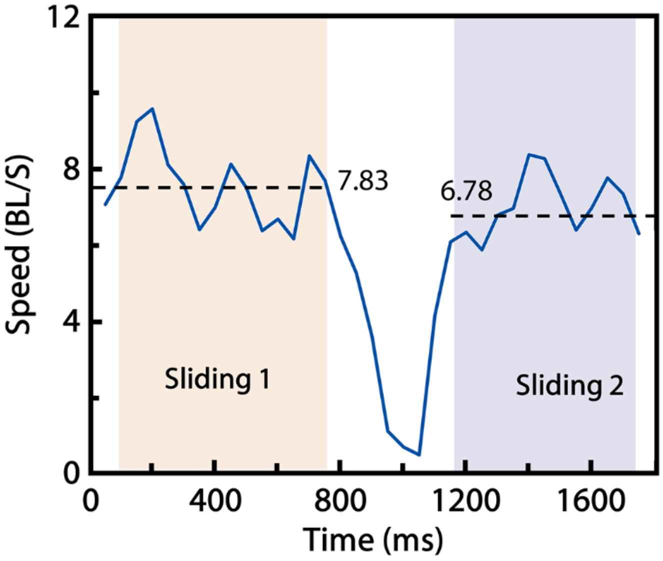

The speed of two fast sliding strokes of a rock-climbing fish on a vertical wall without detaching. Recorded by a high-speed camera with a frame rate of 100 Hz, the fish used in this experiment were 50 mm in body length.

Fig. S2.

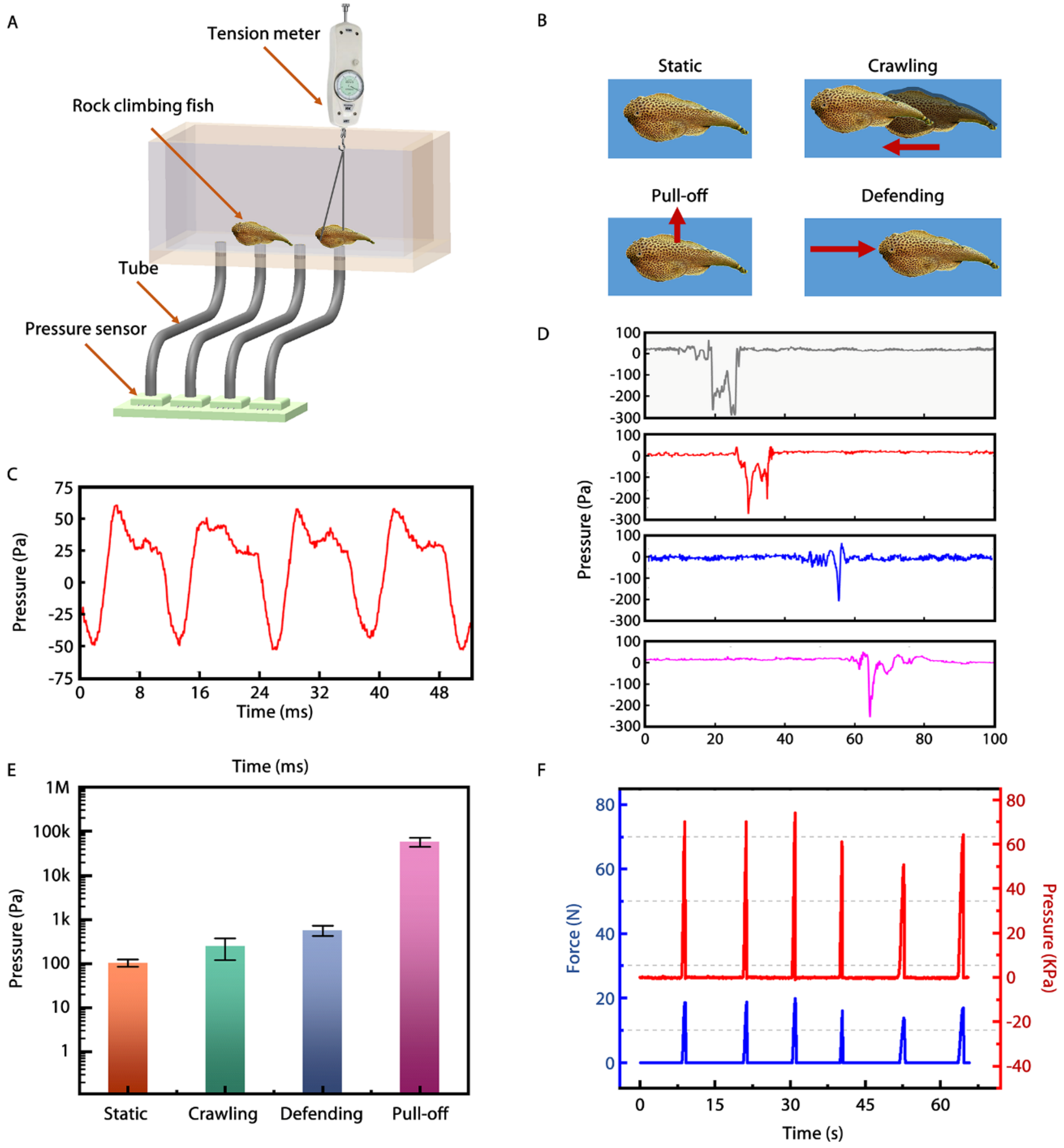

Tunable pressure and adhesion performance of the rock-climbing fish. (A) Experimental setup: Four holes with a diameter of 10 mm were drilled at an interval of 5 cm at the bottom of the water tank, which was bonded with an acrylic plate. The pressure sensors were connected through a catheter, and an oscilloscope was used to collect the signal of the pressure sensor. (B) Definition of four motion states of the fish: the static state, the crawling state, the pull-off state, and the defending state. (C) The suction cup pressure of the fish is in a static adhesion state; the periodic change in pressure may be due to the change in cavity pressure caused by fish breathing. (D) During crawling, four sensors serially recorded the changes in the abdominal pressure of the fish. (E) Negative pressure in the suction cup was measured in different states; the pressure at static, crawling, and defending was acquired with live rock-climbing fish; pull-off pressure was acquired with dead rock-climbing fish. (F) The pull-off force and negative pressure (absolute value) when pulling off a dead rock-climbing fish.

Fig. S3.

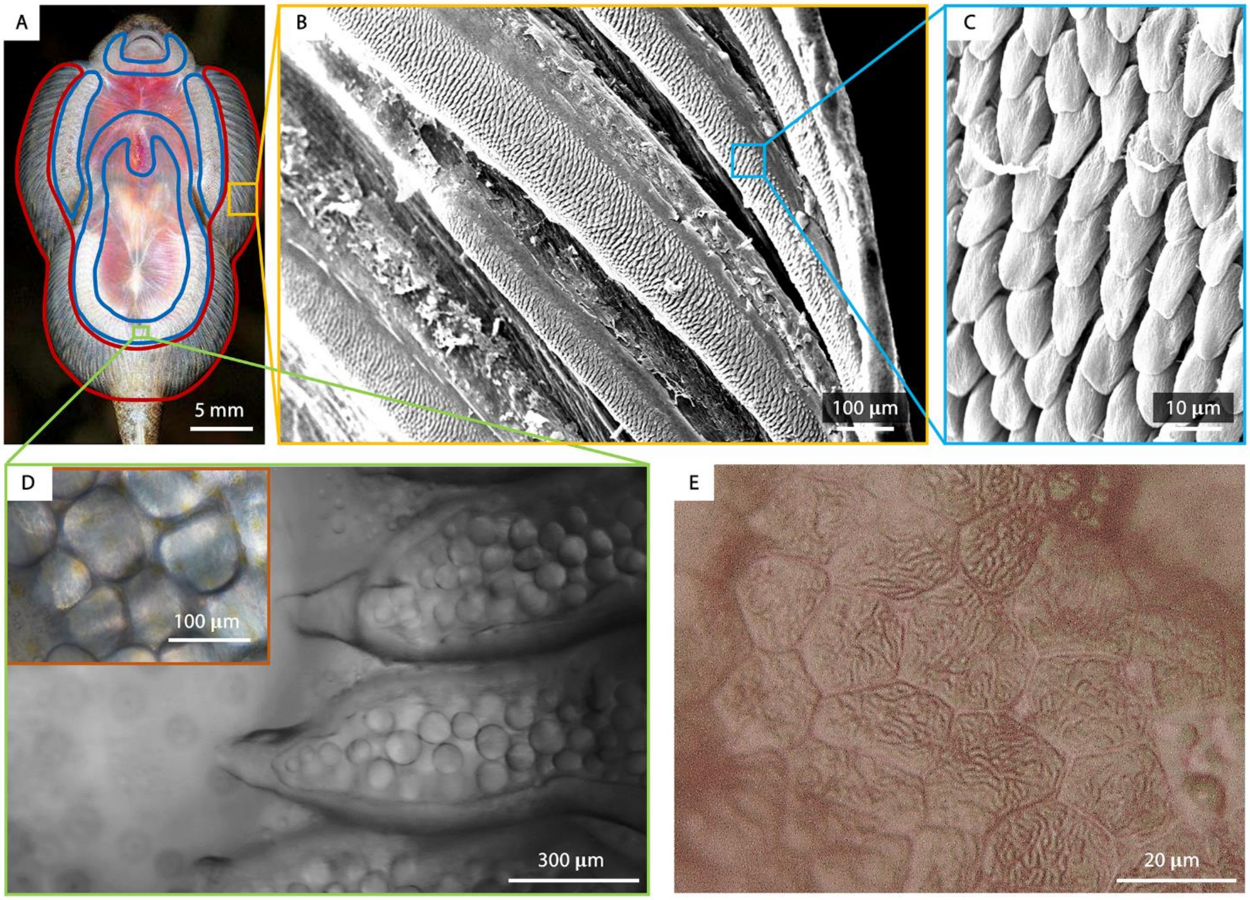

Three microstructures on the fish suction cup. (A) Bottom view of the fish suction cup; (B, C) Setae morphology; (D) Vesicles at a diameter of 20–80 µm; (E) Hexagonal texture structure with a side length of 20–30 µm.

Fig. S4.

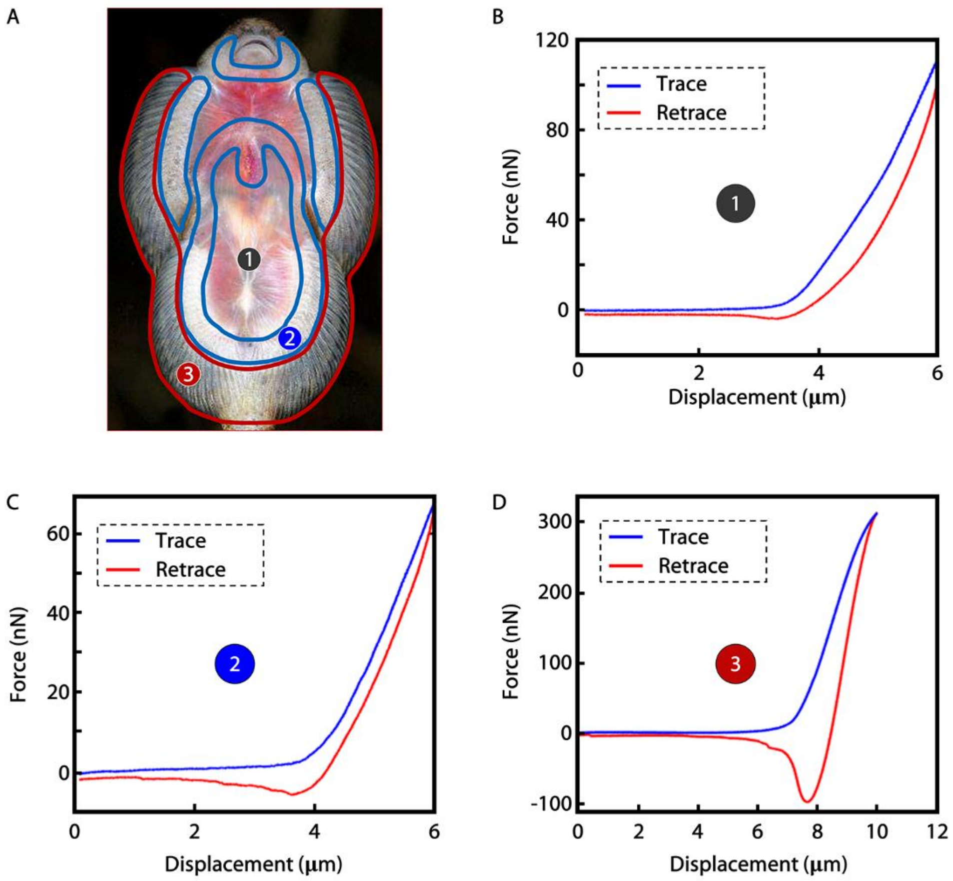

Adhesion force of different regions of the suction disc of the rock-climbing fish. (A) The suction disc is divided into three regions by microstructures. (B) The adhesion force is near zero in the abdomen. (C) A small adhesion force is detected at the junction of the fins and abdomen. (D) The adhesion force reaches 100 nN at setae regions.

Fig. S5.

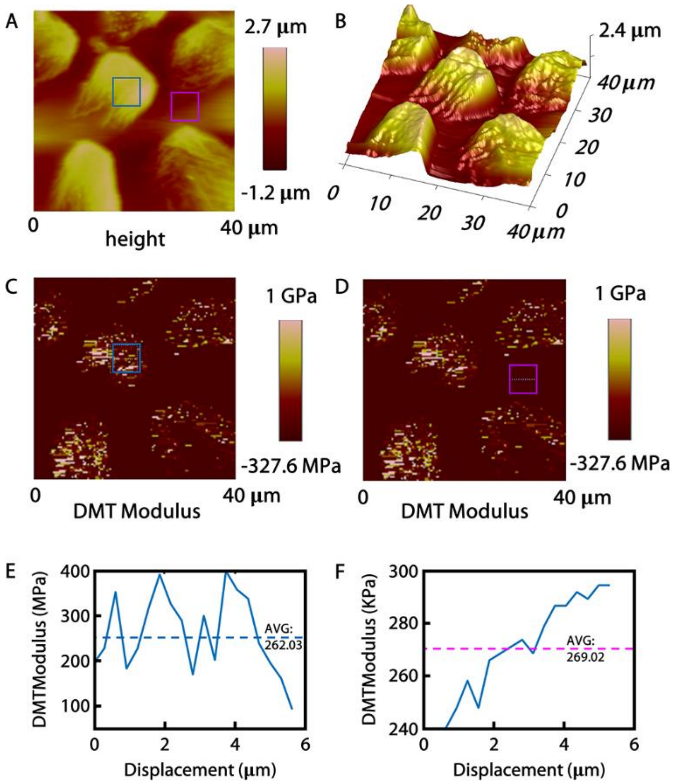

The three-dimensional morphology, and DMT modulus of the micro setae were measured with AFM. (A) Micro setae and tissue height image; (B) Three-dimensional morphology of setae; (C, D) DMT Modulus of micro setae and tissue; (E) the average DMT Modulus of the setae is 262.03 MPa; (F) The average DMT modulus of the tissue is 269.02 KPa.

Fig. S6.

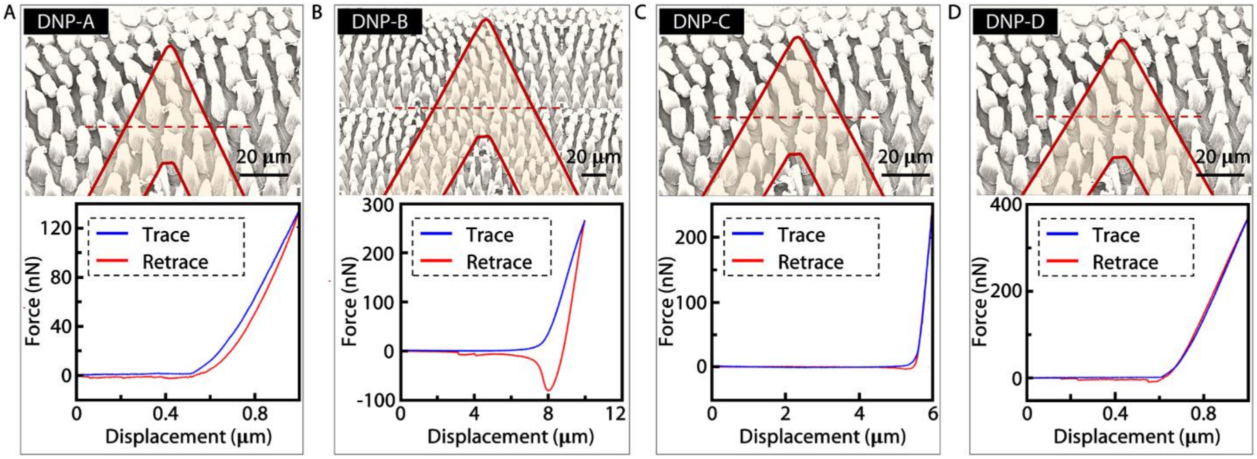

Setae adhesion forces measured with four different sizes of AFM probe cantilevers. (A) The adhesion force of micro setae was measured with a DNP-A cantilever. Micro setae and AFM cantilevers were displayed at the same scale. Under the projected area of the cantilever, approximately four setae were in contact with the probe, and no adhesion force was measured in this situation. (B) The adhesion force of micro setae was measured with a DNP-B cantilever. Under the projected area of the cantilever, approximately eleven micro setae were in contact with the probe. The adhesion force reached 100 nN. (C) The adhesion force of micro setae was measured with a DNP-C cantilever. Under the projected area of the cantilever, approximately five micro setae were in contact with the probe, and no adhesion force was measured in this situation. (D) The adhesion force of micro setae measured with a DNP-D cantilever. Under the projected area of the cantilever, approximately five micro setae were in contact with the probe, and no adhesion force was measured in this situation. Scale bar 20 μm. The geometric dimension parameters of the AFM probe cantilevers are shown in Table S1.

Table S1.

The geometric dimension parameters of AFM probe (DNP) cantilevers

	**Length(µm)**			**Width(µm)**		
	**Nom**	**Min**	**Max**	**Nom**	**Min**	**Max**
**DNP-A**	120	115	125	25	20	30
**DNP-B**	205	200	210	40	35	45
**DNP-C**	120	115	120	20	15	25
**DNP-D**	205	200	210	25	20	30

Table S2.

Statistical data about the crawling and adhesion abilities of different species

Animals	Species	Mass (g)	Max Adhesion (N)	Max Adhesion /mass (times)	Measure situation &Ref.	Body length (mm)	Max Crawling speed (mm/s)	Max BL/S (times)	Measure situation &Ref.
Gecko	Hemidactylus garnoti	NA	NA	NA		46-58	290-770	5.00-16.74	[3]
	Gekko gecko	43.4±1.48	(20.04±1.33)	47.12	[4]	128.9±4.8	600-1200	4.65-9.31	[5]
	Phelsuma dubia	3.8±1.2	(1.670±0.455)	45.97	[6]	42-65	563±103	8.66-13.403	20℃[7]
Clingfish	Gobiesox maeandricus		8.2-42	80-230	[8]	NA	NA	NA	
		1.5-15	7-26 unfouled surface<br>7-18 fouled surfaces	200	[9]	NA	NA	NA	
Tree frog	Kassina maculata	NA	NA	NA		44±4	100-300	2.4-6.8	[10]
	Litoria caerulea	35.23±2.14	0.1-0.9	0.28-2.55	[11]	76.2±3.12	60-140	0.79-1.8	[11]
Remora	Echeneis naucrates	0.24*610	93.4-107.9	63.8-67.1	[12]	NA	NA	NA	
		0.24*284	79	115.9	[13]	NA	NA	NA	
Octopus	Abdopus aculeatus	NA	NA	NA		45	73±21	0.62±0.01	[14]
	Thaumoctopus mimicus	NA	NA	NA		50	51	0.98	[15]
	O.vulgaris	1600(male)<br>476(female)	49.8*8 (male)<br>26.8*8 (female)	24.9<br>45	Single-arm[16]	NA	NA	NA	
Gobiidae	Lentipes concolor	5.02	0.21	136.94	[17]	6.1<br>6.1	10<br>9.9	0.22<br>0.22	[18]<br>[19]
	Sicyopterus stimpsoni	16.6	0.72	125.2	[20]	5.4<br>5.4	9.9<br>7.1	0.17<br>0.19	[19]<br>[18]
Rock climbing fish	Sinogastromyzon puliensis	2.3	29.55	1300	[21]	NA	NA	NA	
	Beaufortia kweichowensis	1.81-2.89	Over 22	1000	[22,23]	60.3 ±3.9	65-103<br>800(escaping)	1.3-2.3<br>16	[22]
		2.4±0.68	23.88±0.68	1015		50	392(sliding)	7.83	This work

Supplementary References

1. Chan DYC and Horn RG. The drainage of thin liquid films between solid surfaces. *The J Chem Phy*1985;**83**:5311–24.

2. Butt H-J and Kappl M. *Surface and Interfacial Forces*. Second Edition. Weinheim: Weinheim Wiley-VCH, 2018.

3. Autumn K, Hsieh ST and Dudek DM *et al.* Dynamics of geckos running vertically. *J Exp Biol* 2006;**209**:260–72.

4. Irschick DJ, Austin CC and Petren K *et al.* A comparative analysis of clinging ability among pad-bearing lizards. *Biological Journal of the Linnean Society* 1996;**59**:21–35.

5. Zaaf A, Van Damme R and Herrel A *et al.* Spatio-temporal gait characteristics of level and vertical locomotion in a ground-dwelling and a climbing gecko. *JOURNAL OF EXPERIMENTAL BIOLOGY* 2001;**204**:1233–46.

6. Bergmann PJ and Irschick DJ. Effects of temperature on maximum clinging ability in a diurnal gecko: Evidence for a passive clinging mechanism? *J Exp Zool Part A-Ecol Integr Physiol* 2005;**303A**:785–91.

7. Bergmann P and Irschick DJ. Effects of temperature on maximum acceleration, deceleration and power output during vertical running in geckos. *J Exp Biol* 2006;**209**:1404–12.

8. Wainwright DK, Kleinteich T and Kleinteich A *et al.* Stick tight: suction adhesion on irregular surfaces in the northern clingfish. *Biol Lett* 2013;**9**:20130234.

9. Ditsche P, Wainwright DK and Summers AP. Attachment to challenging substrates – fouling, roughness and limits of adhesion in the northern clingfish (*Gobiesox maeandricus*). *Journal of Experimental Biology* 2014;**217**:2548–54.

10. Ahn AN, Furrow E and Biewener AA. Walking and running in the red-legged running frog, Kassina maculata. *J Exp Biol* 2004;**207**:399–410.

11. Wang Z, Ji A and Endlein T *et al.* The Role of Fore- and Hindlimbs During Jumping in the Dybowski's Frog (Rana dybowskii). *J Exp Zool Part A* 2014;**321**:324–33.

12. Fulcher BA and Motta PJ. Suction disk performance of echeneid fishes. *Can J Zool* 2006;**84**:42–50.

13. Beckert M, Flammang BE and Anderson EJ *et al.* Theoretical and computational fluid dynamics of an attached remora (Echeneis naucrates). *Zoology* 2016;**119**:430–8.

14. Huffard CL. Locomotion by *Abdopus aculeatus* (Cephalopoda: Octopodidae):walking the line between primary and secondary defenses. *Journal of Experimental Biology* 2006;**209**:3697–707.

15. Hanlon RT, Conroy L-A and Forsythe JW. Mimicry and foraging behaviour of two tropical sand-flat octopus species off North Sulawesi, Indonesia: MIMICRY AND FORAGING BY OCTOPUS. *Biological Journal of the Linnean Society* 2007;**93**:23–38.

16. Margheri L, Laschi C and Mazzolai B. Soft robotic arm inspired by the octopus: I. From biological functions to artificial requirements. *Bioinspir Biomim* 2012;**7**:025004.

17. Maie T and Blob RW. Adhesive force and endurance of the pelvic sucker across different modes of waterfall-climbing in gobiid fishes: Contrasting climbing mechanisms share aspects of ontogenetic change. *Zoology* 2021;**149**:125969.

18. Blob RW, Wright KM and Becker M *et al.* Ontogenetic change in novel functions: waterfall climbing in adult Hawaiian gobiid fishes. *Journal of Zoology* 2007;**273**:200–9.

19. Schoenfuss HL, Maie T and Kawano SM *et al.* Performance across extreme environments: comparing waterfall climbing among amphidromous gobioid fishes from Caribbean and Pacific Islands. *CYBIUM* 2011;**35**:361–9.

20. Baik S, Kim DW and Park Y *et al.* A wet-tolerant adhesive patch inspired by protuberances in suction cups of octopi. *Nature* 2017;**546**:396–400.

21. Chuang Y-C, Chang H-K and Liu G-L *et al.* Climbing upstream: Multi-scale structural characterization and underwater adhesion of the Pulin river loach (Sinogastromyzon puliensis). *Journal of the Mechanical Behavior of Biomedical Materials* 2017;**73**:76–85.

22. Wang J, Ji C and Wang W *et al.* An adhesive locomotion model for the rock-climbing fish, Beaufortia kweichowensis. *Sci Rep* 2019;**9**:16571.

23. Zou J, Wang J, Ji C. The Adhesive System and Anisotropic Shear Force of Guizhou Gastromyzontidae. *Sci Rep* 2016;**6**:37221.
